# Cognitive behavior therapy for autistic adolescents, awareness and care for my autistic traits program: a multicenter randomized controlled trial

**DOI:** 10.1186/s12888-023-05075-2

**Published:** 2023-09-07

**Authors:** Fumiyo Oshima, William Mandy, Mikuko Seto, Minako Hongo, Aki Tsuchiyagaito, Yoshiyuki Hirano, Chihiro Sutoh, Siqing Guan, Yusuke Nitta, Yoshihito Ozawa, Yohei Kawasaki, Toshiyuki Ohtani, Jiro Masuya, Noriko Takahashi, Noriyuki Sato, Shizuka Nakamura, Akiko Nakagawa, Eiji Shimizu

**Affiliations:** 1 Research Center for Child Mental Development, Chiba University, 1-8-1 Inohana, Chuouku, 260-8670 Chiba Japan; 2 Division of Cognitive Behavioral Science, United Graduate School of Child Development, Chiba University, Osaka University, Kanazawa University, Hamamatsu University School of Medicine, Chiba University, University of Fukui, Chiba, Japan; 3 Research Department of Clinical, Educational & Health Psychology, University College London, Gower Street, London, WC1E 6BT UK; 4 Laureate Instituto for Brain Research, 6655 S Yale Ave, Tulsa, OK 74136 USA; 5 Department of Cognitive Behavioral Physiology, Chiba University, 1-8-1 Inohana, Chuouku, 260-8670 Chiba Japan; 6 Biostatistics Section, Clinical Research Center, Chiba University Hospital, Chiba University, 1-8-1 Inohana, Chuouku, 260-8670 Chiba Japan; 7 Safety and Health Organization, Chiba University, Chiba, Japan; 8 Department of Psychiatry, Tokyo Medical University Ibaraki, Medical Center, 3-20-1 3-20-1 Chuo, Ami-machi, Inashiki-gun, Ibaraki, 300-0395 Japan; 9 Fukushima University Child Mental Health-Care Center, 1 Kanayagawa, Fukushima, 960-1296 Japan

**Keywords:** Autism spectrum disorder, Cognitive behavioral therapy, Family therapy, Psychoeducation

## Abstract

**Background:**

Autistic people demonstrate focused interests, sensitivity to sensory stimulation, and, compared with the general population, differences in social communication and interaction. We examined whether a combination of the Awareness and Care for My Autistic Traits (ACAT) program and treatment-as-usual is more effective than only treatment-as-usual in increasing the understanding of autistic attributes, reducing treatment stigma, and improving mental health and social adaptation among autistic adolescents and their parents/guardians.

**Methods:**

Forty-nine adolescents and their parents/guardians were randomly assigned to either a combination of ACAT and treatment-as-usual or only treatment-as-usual. The combined group received six weekly 100-minute ACAT sessions, while the treatment-as-usual group received no additional intervention. The primary outcome was the change in understanding of autistic attributes (Autism Knowledge Quiz-Child), administered from pre- to post-intervention. The secondary outcomes included the change in Autism Knowledge Quiz-Parent, reduced treatment stigma, and improved mental health and social adaptation among autistic adolescents and their parents/guardians. A primary outcome measure scale was scored by assessors who were blind to the group assignment.

**Results:**

The combined group (both autistic adolescents and their parents/guardians) showed an increase in Autism Knowledge Quiz scores compared to those in the treatment-as-usual group. Autistic adolescents in the combined group also demonstrated a decrease in treatment-related stigma and an improvement in general mental health compared to those in the treatment-as-usual group, while there were no group differences in the change in social adaptation. For parents/guardians, there were no group differences in the change in treatment-related stigma, general mental health, adaptive skills, or attitudes toward their children.

**Conclusions:**

The ACAT program could be an effective treatment modality to increase the understanding of autistic attributes among both autistic adolescents and their parents/guardians. The ACAT program positively affects self-understanding, reduces treatment stigma, and stabilizes behavioral issues for autistic adolescents as a part of mental health measures, but it does not effectively reduce treatment barriers or improve mental health for parents/guardians. Further research should consider whether additional support for parents/guardians could be beneficial.

**Trial registration:**

: The study was registered in UMIN (UMIN000029851, 06/01/2018).

**Supplementary Information:**

The online version contains supplementary material available at 10.1186/s12888-023-05075-2.

## Background

### Autistic people and their mental health

Autistic people demonstrate focused interests, sensitivity to sensory stimulation, and, as compared with the general population, differences in social communication and interaction [1]. We use “autistic” as an “identity-first” term for autism spectrum disorder [2]. Autistic traits can make adolescents prone to interpersonal and academic difficulties in school [3]. Moreover, in response to stress or changes in routine, autistic adolescents may experience behavioral problems such as aggression, withdrawal, self-stimulation, and self-harm [4]. As autistic individuals become older, they experience increased rates of depression and anxiety as compared to non-autistic individuals [5]. For example, an additional comorbid mental disorder is observed in 42% of young autistic people—two to four times higher than in their typically developing counterparts [6]. There are many factors contributing to the development of comorbid mental disorders in autistic individuals. One such factor is treatment stigma, which includes prejudice and discrimination against receiving treatment and support [7]. Treatment stigma impedes help-seeking behavior, which can worsen the prognosis of autistic individuals. For example, because of treatment stigma, autistic adolescents may refrain from requesting and using reasonable accommodations/services to which they are entitled [8], which may lead to social isolation and maladjustment.

### Psychological interventions for improving autistic individuals’ mental health

Various interventions are used to treat social maladjustment and comorbid psychiatric disorders in autistic individuals. For example, psychotherapy, such as cognitive-behavioral therapy (CBT) and social skills training, can be administered to improve coping skills and environmental adjustment [9]. However, there are very few interventions aimed at improving self-awareness and reducing treatment stigma in autistic adolescents. Stigma can affect autistic children and their parents/guardians. Parents/guardians of autistic children often face stigmatizing attitudes from others, which increases treatment stigma toward their children [10].

### Potential of psychological interventions to enhance autistic self-perception

Stigma against autism among parents/guardians may prevent autistic adolescents from receiving reasonable accommodations, which in turn, may increase their psychological burden. Destigmatizing the attitudes of parents/guardians is important to lower autistic individuals’ treatment stigma as parents/guardians can help children perceive their autistic characteristics more positively through open discussions [11]. In Riccio et al.’s [11] study, most autistic adolescents were unaware of their diagnosis or did not have sufficient knowledge about their condition. Based on interviews and observations of verbal exchanges between autistic adolescents and their mothers, the researchers reported that adolescents whose parents voluntarily disclosed their diagnosis to them were better able to define their autistic traits compared to adolescents whose parents told them about their autism involuntarily or not at all. They also reported that autistic adolescents had a harder time defining their autism than did their guardians; therefore, guardians could help children perceive their autism more positively by discussing it early in their development. Thus, learning about one’s autism diagnosis and increasing awareness of autistic traits could help autistic adolescents understand their strengths, which may reduce treatment stigma.

Importantly, Riccio et al. [11] reported that some autistic teens, particularly those whose diagnosis was not disclosed voluntarily by their parents, viewed autism in stigmatizing ways and struggled to reconcile the diagnosis with their sense of self. This indicates that conversations about autistic characteristics between children and parents can facilitate children’s awareness and understanding of autistic traits. However, treatment stigma toward autism among parents/guardians may prevent them from talking to their children about their diagnosis and characteristics. One strategy to reduce parents’ treatment stigma is to increase their understanding of their child’s autistic traits [12, 13]. Therefore, joint family psychoeducation programs for both adolescents and parents are necessary to increase adolescents’ and parents’ understanding of autistic characteristics, rather than treating only autistic adolescents as patients, to reduce treatment stigma [14].

### Psychoeducation program to improve self-understanding of autistic traits

The Psychoeducation Group for Autism Spectrum Understanding and Support (PEGASUS) in the UK is one of the few evidence-based, structured family psychoeducation programs for autistic traits worldwide [14]. However, despite showing promise for increasing autism knowledge and self-awareness in both autistic young people and their parents, it did not reduce adolescents’ mental health issues and improve self-esteem in a clinical trial [14]. The insufficient effect of PEGASUS on mental health issues and self-esteem is understandable as it is a psychoeducation program and does not incorporate strategies to target autistic traits related to other problems, such as mental health issues, stigma, or social adaptation.

### Development of a new program on awareness and coping strategies for autistic traits using CBT

CBT is an evidence-based treatment for children, adolescents, and adults who have mental health issues such as depression and anxiety. It can be modified and applied to treat mental health issues in autistic children [15, 16]. CBT aims to help recipients become aware of their negative interpretations and behavioral patterns that reinforce distorted thinking. CBT uses a combination of cognitive and behavioral problem-solving skills to help people understand the settings and situations that cause issues, and then implement those skills in their daily lives. Therefore, we developed a family psychoeducation CBT program for autistic adolescents and their families—the Awareness and Care for My Autistic Traits (ACAT) [17]. Through the psychoeducation component of the ACAT program, we aim to increase knowledge and awareness regarding autistic traits to avoid bias against treatments, while the CBT component is meant to improve adolescents’ coping skills and their implementation with the support of parents/guardians. Altogether, the ACAT program is expected to promote awareness of the strengths and weaknesses of individual autistic characteristics, teach step-by-step reasonable accommodation and coping strategies for maladaptive behaviors, and improve the social and psychological adjustment of autistic adolescents and their parents/guardians. The contents of the ACAT program and its differences from an existing psychoeducational program, the PEGASUS, are displayed in Table 1.

**Table 1:** Similarities and differences between the PEGASUS and ACAT program

	PEGASUS	ACAT
Intervention type	Group; up to six young people and their parents	Individual; a one-on-one session consisting of a therapist and a child-parent/guardian pair
Intervention style	Parallel, a child and their parent/guardian attend intervention separately	Co-psychoeducation with a child and their parent/guardian
Intervener	Two facilitators	One therapist
Aims	Increasing the awareness of a child’s autistic traits and learning compensation strategies	Increasing the recipient’s awareness of a child’s autistic traits Understanding the child’s autistic responses Practicing problem-solving skills
Frequency of intervention	One session/week for six sessions Each session lasts 90 min	One session/week for six sessions Each session lasts 100 min Additional sessions before the first session and follow-up session
Age range (for children)	9–14 years	10–17 years
Intervention method	Group discussion Children’s group: After attaining general knowledge about autistic traits, the group discusses the strengths, weaknesses, and characteristics of children’s autistic traits in the unit “autistic traits and me. They also discuss compensation strategies for autistic traits. Parents’ group: The group reflects on the impact of autistic traits on their family and the surrounding environment. They also discuss potential resources and networks around the child and family.	Co-psychoeducation and CBT 1. Psychoeducation on autistic traits 2. Using the CBT model 3. Discuss problem-solving methods and plan what kind of problem-solving strategies will improve the child’s adjustment. See Table 2 for details.

The objective of this study was to evaluate the effectiveness of the ACAT intervention compared to treatment-as-usual (TAU). The specific hypotheses were as follows:

1. Adolescents and parents/guardians assigned to the combined ACAT and TAU (COMB) group would show improved Autism Knowledge Quiz (AKQ) scores compared to those assigned to the TAU group alone.
2. Adolescents assigned to the COMB group would show reduced treatment stigma and improved mental health, including coping strategies and social adaptation, compared to those in the TAU group.
3. Parents/guardians assigned to the COMB group would show an increased awareness of children’s autistic traits and improved mental health and adaptive skills to deal with their children’s challenges compared to those in the TAU group.

## Methods

This study was designed as a prospective, randomized, and assessor-blinded endpoint trial with two parallel intervention groups—COMB and TAU—consisting of a six-week intervention for both groups conducted as a multisite study.

We evaluated outcomes at pre-intervention (0 weeks), post-intervention (6 weeks), and follow-up (10 weeks). The Chiba University Research Ethical Review Committee approved this trial (CRB3180015, No. G29027). All procedures were carried out in accordance with relevant guidelines and the regulations of the Declaration of Helsinki.

### Randomization

Participants were allocated to either the intervention group (ACAT + TAU) or control group (TAU + waiting list to intervention) in a 1:1 ratio using the minimization method, based on gender (boy, girl, or other) and awareness of their autistic traits by the AKQ-Child (AKQ-C) (total score more or less than 2.4) at baseline. The randomization procedure was conducted by Chiba University Hospital’s independent data management team using a computer program. Participants allocated to the control group were informed that they could receive the ACAT program after this randomized controlled trial was completed. Therapists and participants were not blinded to their group assignment; however, two trained testers, MH and MS, who assessed only the AKQ-C and AKQ-Parent (AKQ-P), were blinded to the groups.

### Participants

The adolescents and parents/guardians provided written and verbal informed consent using the form and protocol approved by the Chiba University Clinical Research Ethical Review Committee. The inclusion criteria for adolescents were: (1) diagnosed with autism by their general practitioners using the Diagnostic and Statistical Manual of Mental Disorders, Fifth Edition, and met the cut-off value for autism measured using the Autism Diagnostic Observation Schedule (ADOS-2) [18] and Autism Diagnostic Interview-Revised (ADI-R) [19, 20]; (2) had a verbal intelligence quotient (IQ; Verbal Comprehension Index [VCI]) of ≥ 90 as measured by the Wechsler Adult Intelligence Scale-III or Wechsler Intelligence Scale for Children, Fourth Edition (WISC-IV) ([21]); (3) had a social difficulty assessment of moderate and above, as measured using the Strengths and Difficulties Questionnaire (SDQ; Goodman [22]); (4) were aged between 10 and 17 years; (5) were outpatients at a psychiatric clinic/hospital. The inclusion criterion for parents/guardians was that they lived with the autistic individual. This study was conducted at a clinic/hospital; thus, all participants visited the hospital’s psychiatry department. We chose a VCI of ≥ 90 as the recommended IQ for providing CBT for autistic children is typically approximately 70–100 [16].

The exclusion criteria for autistic adolescents were: (1) suicidal intentions at the time of enrolment; (2) a repetitive behavioral problem and/or severe degenerative physical disorder, as identified by a primary psychiatrist; (3) any other condition that might compromise well-being. The exclusion criterion for parents/guardians was meeting the diagnostic criteria for psychiatric disorders, assessed using the Mini-International Neuropsychiatric Interview (M.I.N.I.) [23]. We excluded parents/guardians with psychiatric disorders as they would not have been able to provide adolescents with the required support.

### Sample size

In a previous study by Gordon et al. [14], the change in autism awareness on the AKQ-C, the primary outcome in this intervention, was 2.26 with an effect size of 0.92. Based on the above report, we used G*Power 3.1.9.2 for detection. For a significance level of 5% on both sides and a power of 80%, 20 cases per group were required. Considering dropouts and 20% ineligible cases, the required number of cases was set at 24 per group, for a total of 48 cases.

### Intervention groups

#### COMB group

The ACAT program is an individual CBT intervention conducted once a week for 100 min each time [17]. It encompasses six sessions, starting with feedback on the results of psychological assessments (i.e., ADOS-2, ADI-R, ADHD-RS, Sensory Profile, and WISC-IV) related to autistic traits at baseline and ending with a follow-up session one month after the completion of six sessions. The ACAT program consists of three main phases (Table 2). The first step involves helping autistic individuals verbalize and attain metacognitive awareness of their autistic traits. This way, they practice “noticing” their autistic traits in daily life. The second step aims to understand their autistic responses to environmental or social stressors by writing down their thoughts, feelings, and behaviors using the CBT model (Figure S1). Through this process, autistic individuals and their parents/guardians can understand how autistic traits affect their daily lives. In the third stage, autistic individuals write down coping strategies they think they can use and try to apply strategies in their daily lives. Furthermore, an adolescent, their parent/guardian, and a therapist discuss what kind of reasonable accommodations could be implemented to improve the adolescent’s adjustment and plan how they could implement them in daily life (Figure S2). The therapists were certified clinical psychologists (which requires a master’s degree in Japan), specialized in autistic adolescents, and with more than three years of psychotherapy experience in this area. The two interviewers of the primary outcomes were master’s-level graduate students.

**Table 2:** Outline of ACAT components across the six-week program

Session	Themes ο Session content	Session goals ο Required CBT techniques
Pre-session	Learn about your psychological assessment for autism ο Sharing the results of psychological assessment tests ο Learn about ASD/ADHD ο Learn about the aims of the ACAT program. ο Outline the current problem	Learning about their characteristics and autistic traits through the results of assessment tests ο Monitoring ο Externalization
1st CBT	Learn about the characteristics of your autistic traits ο What is social and psychological adaptation? ο What is CBT? ο To understand the link between autistic traits and maladaptive problems	Understanding the differences between autistic attributes and typical developmental features ο Monitoring ο Externalization
2nd CBT	Cognitive changes: Learn not only about the “weakness” but also the “strengths” of your autistic traits ο To understand the strengths and weaknesses of autistic traits ο To understand the link between autistic traits and maladaptive problems based on the cognitive behavioral model	Discovering the “strengths” of autistic traits, setting aside the “weaknesses” and turning negative perspectives into rational neutral understanding about themselves. ο Monitoring ο Externalization ο Case formulations
3rd CBT	Cognitive changes: Create “funny nicknames” for your autistic traits. οNaming the autistic traits οTo understand the link between autistic traits and maladaptive problems based on the cognitive behavioral model	Learning to externalize their autistic characteristics, facilitate self-monitoring skills, and develop metacognition. ο Monitoring ο Externalization ο Case formulations
4th CBT	Behavioral changes: Create a problem-solving plan for your autistic traits ο Case formulations in CBT models to identify patterns of maladaptive problems	Learning how they can change and adjust problematic behaviors to cope with their autistic traits ο Case formulations
5th CBT	Behavioral changes: Plan a functional way to solve easy problems that are caused by your autistic traits with your parents ο Plan and practice coping strategies (cognitive and behavioral changes: reasonable accommodation) based on the CBT model	Make problem-solving plans for their autistic traits and will also consider how to cope with problems when they execute their plans. Starting with easier problems related to their autistic traits. Conducting these plans as a homework assignment. ο Problem-solving techniques
6th CBT	Behavioral changes: Plan a functional way to solve more complicated problems that are caused by your autistic traits with your parents ο Plan and practice coping strategies (cognitive and behavioral changes: reasonable accommodation) based on the CBT model ο A summary of what we have done up to now	Make problem-solving plans for their autistic traits and will also consider how to manage more complicated problems when they execute their plans. Conducting these plans as a homework assignment (this homework assignment will be checked at a follow-up session). ο Problem-solving techniques
CBT follow-up	Check how well you are living with your autistic traits ο Review what you have done in the ACAT program ο Identify patterns of future coping strategies and develop a perspective on coping after the session	Reviewing all skills and techniques learned to date. Check their understanding of their autistic traits and discuss the progress made in therapy, areas of continued effort, and ongoing challenges. ο Monitoring ο Case formulations ο Problem-solving techniques

#### TAU

The participants in the TAU group received no intervention from the research team. TAU involved regular 10-minute counseling sessions with a psychiatrist and, in some cases, medication and other coping therapies.

### Assessment and outcomes

#### Participant characterization

##### ADI-R

The ADI-R [19, 20] is a structured parent interview used to identify a clinical impression of autism. The ADI-R has excellent reliability and validity for the diagnosis of autism. All examiners were trained to reliability standards for administration and scoring (i.e., > 80% agreement with a reliable coder, F.O., who has a research license for the ADI-R).

##### ADOS-2

The ADOS-2 [18] is a semi-structured, observational assessment of communication, social interaction, and imaginative play. It is used with adolescents and maps directly onto diagnostic criteria for autism. Algorithm scores for communication and socialization are calculated to support a diagnosis of autism. All examiners were trained to reliability standards for administration and scoring (i.e. > 80% agreement with a reliable coder, F.O., who has a research license for the ADOS-2).

##### WISC-IV

The WISC-IV [21] is a widely used representative intelligence test for children/adolescents. It consists of 15 subtests, and by administering the 10 basic tests, five composite scores are calculated by psychologists with a minimum of a master’s-level qualification and extensive experience.

##### M.I.N.I.

The MINI [23] is used to assess 17 Diagnostic and Statistical Manual of Mental Disorders Fourth Edition Axis I disorders and is a well-validated instrument. In this study, it was used to confirm the presence or absence of diagnosis of psychiatric disorders in parents/guardians by psychologists with a minimum of a master’s-level qualification and extensive experience.

#### Adolescents’ outcome measures

##### Primary outcome

AKQ-C. The English AKQ-C [14] was translated into Japanese by a bilingual postgraduate student. The Japanese version was further back-translated by English native speakers who are also fluent in Japanese. The author of the English AKQ-C confirmed that the contents of the back-translated and original tool were consistent. The AKQ-C has two sections. The first consists of a five-item structured open-ended interview on “awareness of autistic characteristics,” designed to measure self-knowledge and strengths and difficulties associated with autistic traits. The second consists of a 15-item general knowledge quiz for autistic traits and includes questions on the nature, prevalence, and hypothesized underlying neurobiological causes of autism, as well as the strengths and difficulties commonly observed in autistic people. We only used the first section of the AKQ-C, the index of awareness of autistic characteristics, as a primary outcome as the ACAT program was developed to increase the understanding of one’s autistic traits rather than improve general knowledge of autistic traits. The details of the scoring of AKQ-C Sect. 1 are explained in the supplemental material. Briefly, an assessor asked an autistic adolescent about their strengths and weaknesses, scoring each descriptive response in a binary manner (1 as related to autistic traits, and 0 as not related to autistic traits). The assessor was blinded to the group assignment, and scoring was supervised by WM.

The AKQ-C was administered to autistic adolescents at pre-intervention (week 0: before starting the first session), post-intervention (week 6: when they completed six sessions), and follow-up (week 10: one month after the completion of sessions). The change in AKQ-C scores from pre- to post-intervention was considered the primary outcome.

##### Secondary outcomes

As secondary outcomes for adolescents, self-report and parent/guardian-report questionnaires and semi-structured interviews for parents were used, and as secondary outcomes for parents/guardians, only self-report questionnaires were used to assess the efficacy of the interventions.

Barriers to Access to Care Evaluation scale version 3. The Barriers to Access to Care Evaluation Scale version 3 (BACE-3) [24] is a self-report 30-item questionnaire that assesses barriers to mental health care for people with mental health problems. Each item is scored from 0 (*not at all*) to 3 (*a lot*). The BACE-3 comprises two subscales: “treatment-related stigma” (12 items) and “treatment-unrelated stigma” (18 items). We used only the “treatment-related stigma” subscale. A higher score indicates a greater barrier to treatment-seeking behaviors. The Japanese version of the BACE-3 was examined for reliability and validity in people with mental health problems aged 20 to 65. Cronbach’s alpha for the treatment stigma subscale was 0.90, indicating good internal consistency [7]. Adolescents in this study responded to the BACE-3 at three time points: pre-intervention, post-intervention, and follow-up.

Depression Self-Rating Scale for Children. The Depression Self-Rating Scale for Children (DSRS-C) [25] is an 18-item instrument, the reliability and validity of which have been demonstrated worldwide. Each item is scored on a three-point scale: “*always*,” “*sometimes*,” and “*never*. A higher score indicates increased severity of depression. It can be applied from childhood, from ages 7 to 13 [25]. However, in several studies, it has been used for ages 14 to 18 [26, 27]. Autistic adolescents in this study answered the DSRS-C at three time points: pre-intervention, post-intervention, and follow-up.

SDQ. The SDQ [21, 28] is a parent-rated short screening instrument that addresses the general mental health of adolescents. It includes 25 items, each of which can be marked as “not true,” “somewhat true,” or “certainly true. A higher score indicates more severe multifaceted behavioral and mental problems. The target age range is 4–18 years. The SDQ was completed by parents/guardians at three-time points: pre-intervention, post-intervention, and follow-up.

Vineland Adaptive Behavior Scales Second Edition. The Vineland Adaptive Behavior Scales Second Edition (Vineland-II) [29] is a semi-structured interview for parents/guardians to capture the developmental norm of social adaptive behavior in individuals aged 0–92 years. The Vineland-II has excellent reliability and validity [29]. Independent assessors interviewed parents/guardians about their adolescents at three time points: pre-intervention, post-intervention, and follow-up.

#### Secondary outcomes for parents/guardians

##### AKQ-P

Like the AKQ-C [14], the English AKQ-P was translated into Japanese and validated. We also used only the first section, “awareness of autistic traits,” to measure awareness of adolescents’ strengths and difficulties related to their autistic traits. The AKQ-P replaces “you are” with “your child is” but all other aspects are the same as on the AKQ-C. The scoring for the AKQ-P is the same as that for the AKQ-C. Parents/guardians responded to the AKQ-P at three time points: pre-intervention, post-intervention, and follow-up.

##### BACE-3

Similar to the assessment of adolescents, the BACE-3 was administered to their parents/guardians at three time points: pre-intervention, post-intervention, and follow-up.

##### General health questionnaire

The General Health Questionnaire 12 (GHQ-12) [30] is used to measure current mental health and psychological distress. Each of the 12 items is rated on a four-point scale (l*ess than* usual, *no more than usual*, *more than usual*, or *much more than usual*). A higher score indicates greater psychological distress. The target age is 12 years and above. Parents/guardians responded to the GHQ-12 at three time points: pre-intervention, post-intervention, and follow-up.

##### Parenting resilience elements questionnaire

The Parenting Resilience Elements Questionnaire (PREQ) [31] measures the degree to which parents are “resilient” in adapting to the challenges and difficulties associated with children with developmental disabilities. It consists of 29 items across three factors: “knowledge of child characteristics,” “awareness of social support,” and “positive perceptions of parenting. Each item is scored on a seven-point Likert scale ranging from 1 (*strongly disagree*) to 7 (*strongly agree*). Higher scores on each item indicate greater resilience in dealing with child-related challenges and difficulties. Parents responded to the PREQ at three time points: pre-intervention, post-intervention, and follow-up.

#### Other measures

Adherence to the ACAT manual and CBT were measured using the Revised Cognitive Therapy Scale (CTS-R) [32]. The CTS-R score ranges from 0 to 6, and a score of ≥ 4 indicates proficiency in CBT [32]. A researcher sat with the therapist and listened to each session to rate if the CTS-R was consistent with the ACAT textbook.

### Recruitment and participant flow

From March 2018 to August 2020, recruitment, treatment, and data collection were conducted in Chiba and Fukushima, Japan. Adolescent outpatients (10–17 years) had been diagnosed with autism using gold-standard instruments such as the ADOS-2 and ADI-R. Their parents/guardians were informed about the intervention in the waiting rooms at Chiba University Hospital and a local psychiatry clinic. For those who showed interest, more information was provided by the recruitment staff (FO and NT), and parents/guardians of prospective research participants contacted the researchers either by phone or email address.

**Fig. 1:**
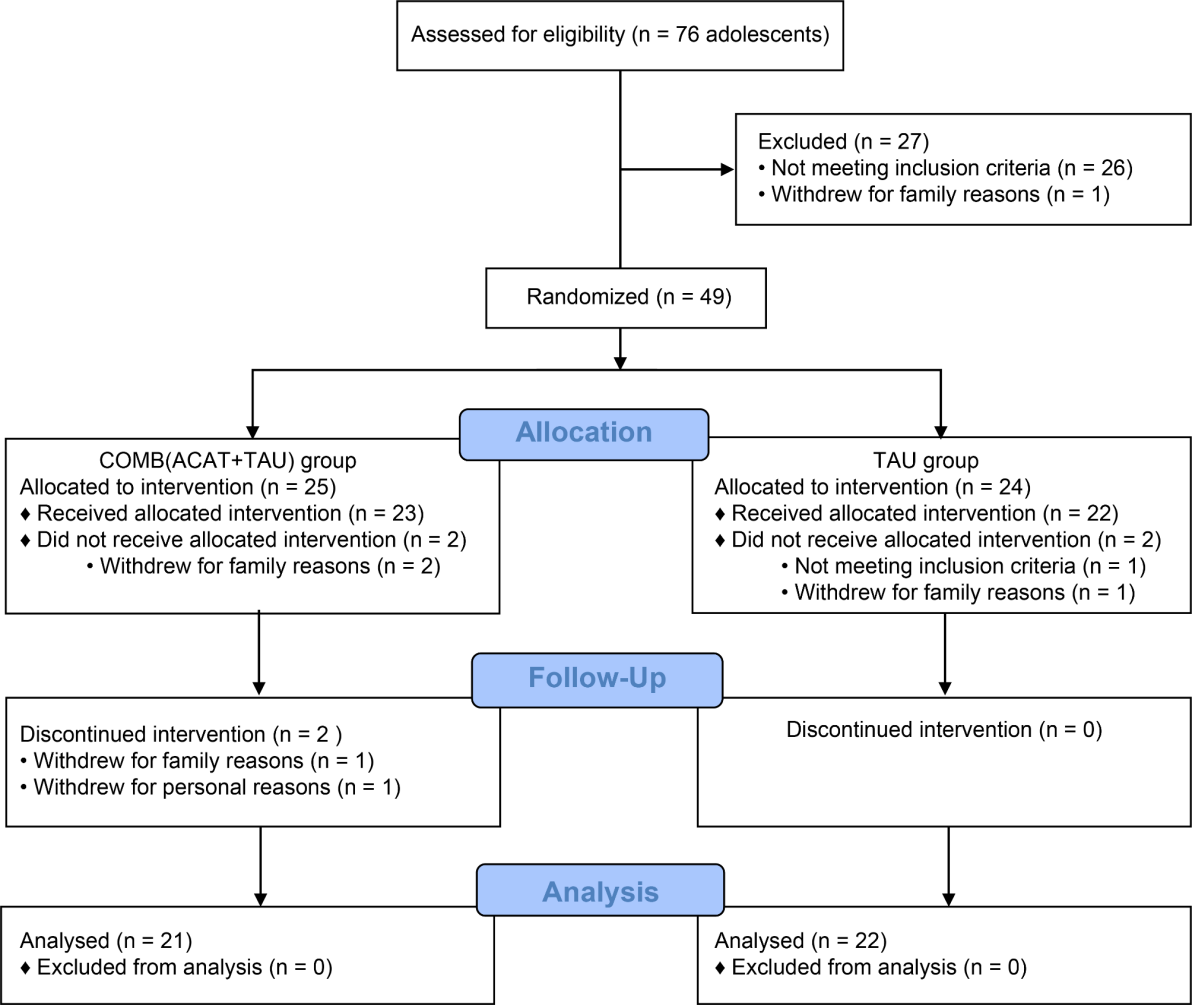
The flow of participants through the trial

The CONSORT flow of participants throughout the trial is shown in Fig. 1. A total of 76 potential participants were assessed for edibility, and 27 were excluded because they did not meet the inclusion criteria (12 autistic adolescents and five parents/guardians), met the exclusion criteria (nine autistic adolescents), or withdrew for family reasons before participation (one autistic adolescent). After the assessment for eligibility, 49 participants were randomly allocated to the intervention group (n = 25) or the control group (n = 24). In the intervention group, two participants withdrew during the intervention and two at follow-up for family or personal reasons. Thus, data from 21 participants were used for analysis. As for the control group, one participant withdrew for family reasons and one was found not to meet the inclusion criteria during the intervention; therefore, data from 22 participants were analyzed.

### Statistical analyses

All statistical analyses were performed as pre-registered [17]. The primary outcome was the AKQ-C score, and we examined differences in score variations from baseline (week 0) to post-intervention (week 6) between the COMB and TAU groups, with allocation factors (i.e. AKQ-C scores and gender) as covariates (analysis of covariance). The differences in score variations from baseline (week 0) to follow-up (week 10) between the two groups were the secondary outcome. Other secondary analyses included psychometric variables of adolescents and parents/guardians (described above), and we evaluated the effect size of Cohen’s d and longitudinal data on differences in scores from week 0 to week 6 and week 0 to week 10 between the two groups. Student’s *t*-tests were used to compare the differences between the two groups. The primary outcome was the change in the score at 6 weeks on the AKQ-C, with TAU vs. COMB comparisons by *t*-test. The secondary outcome was a TAU vs. COMB comparison of the Vineland-II, SDQ, DSRS-C, and BACE-3 child versions by *t*-test. The significance level was set at 5% for the primary analysis. In the secondary analysis, the significance level was set at 0.01 to allow for multiplicity adjustment by a total of five primary and secondary outcomes by Bonferroni correction. Cohen’s (1988) recommendations stipulate that a value > 0.20 denotes a small effect, > 0.50 signifies a medium effect, and > 0.80 indicates a large effect. All analyses were performed using SAS version 9.4 (SAS Institute, Cary, NC, USA).

## Results

The demographic information for participants and parents/guardians in each group is provided in Table 3. Throughout the study, all participants in the COMB and TAU groups received medical care from psychiatric hospitals. The male-to-female ratio of the participants was 33:12, with more men than women. The average age when starting the program was 13.5 years, and the average age of diagnosis of autism was 10.3 years. Detailed information regarding demographics is provided in Table 3.

**Table 3:** Characteristics of the participants

		Whole sample	TAU group	COMB group
		n = 45	n = 22	n = 23
Males/females		33, 12	17, 5	16, 7
Mean age in years (*SD*)		13.5 (2.2)	14.1 (2.0)	12.8 (2.2)
Age range in years		10 to 17	11 to 17	10 to 17
School attendance				
	1. Everyday	29	14	15
	2. School refusal of more than 1/3	14	6	8
	3. Not student	2	2	0
Mean years of schooling (*SD*)		8	8.6	7.3
Range of years of schooling		4–12	5–12	4–12
Support or accommodation in school or at the workplace				
	1. Special class	5	1	4
	2. Special support services in resource rooms	2	0	2
	3. Reasonable accommodation	2	2	0
	4. None	36	19	17
Mean age at diagnosis of ASD in years (*SD*)		10.3 (4.1)	10.4 (4.9)	10.2 (3.3)
Age range at diagnosis of ASD in years		2–17	2–17	4–15
Past psychiatric history		14	7	7
Diagnosed with other neurodevelopmental disorders		19	10	9
	1. ADHD	16	9	*7*
	2. LD	4	1	3

ADHD, attention-deficit/hyperactivity disorder; ASD, autism spectrum disorder; LD, learning disorder.

Of the guardians participating in this study, 44 were mothers and one was a father. Their average age was 45.7 years, with an average of 14.3 years of education, and 14.4% were in full or part-time employment. Eight of the 44 participants had received support services such as parent training. Detailed information regarding these demographics is provided in Table 4.

**Table 4:** Characteristics of guardians

			Whole sample	TAU group	COMB group
			n = 45	n = 22	n = 23
Guardian					
	1. Father		1	0	1
	2. Mother		44	22	22
	3. Other		-	-	-
Mean age in years (*SD*)			45.7 (5.4)	46.6 (4.5)	44.8 (6.1)
Mean years of schooling (*SD*)			14.3 (1.6)	14.6 (1.4)	14.1 (1.8)
Mean years of employment (*SD*)			14.4 (8.2)	12.7 (8.4)	16 (7.8)
Employment			28	11	17
	Employment status				
	1. Full-time		13	5	8
	2. Part-time		15	6	9
	Reason for unemployment				
	1. Student		-	-	-
	2. Homemaker		17	11	6
	3. Temporary retirement		-	-	-
Support for guardian			8	3	5
Psychiatric history			9	4	5
Household income (million yen_					
	< 4		3	2	1
	4–5		3	2	1
	5–6		-	-	-
	6–7		8	3	5
	7–8		7	5	2
	8–9		10	5	5
	9–10		6	1	5
	10–20		8	4	4
	≥ 20		-	-	-

### Adherence to the ACAT manual and CBT

All ACAT activities were performed according to the manual [17]. To measure adherence to the ACAT manual and CBT, 12 randomly selected sessions out of 270 (six session times, 45 dyads) were rated by a researcher who had not been involved in program development (Table 1). The average CTS-R score across each session was 4.2 (standard deviation [SD] = 0.8). Moreover, we did not observe any critical harm or unintended effects in each group. Therefore, adherence to the ACAT manual and CBT was ensured to some extent.

### Baseline characteristics

The demographic characteristics of the COMB and TAU groups are summarized in Tables 3 and 4. There were no significant differences in demographic characteristics at baseline, except for the participants’ age of consent (COMB: 12.8 years, TAU: 14.1 years, *p* = .04) (Table 3). The primary outcome, the AKQ-C score, did not differ significantly between the two groups at baseline. In addition, there were no significant differences in the scores on the BACE-3, DSRS-C, SDQ, and Vineland-II between the two groups for adolescents, and in the scores on the BACE-3, GHQ-12, and PREQ between the two groups for parents/guardians at baseline (Table 5).

**Table 5:** Outcomes at baseline, post-intervention, and follow-up

		Baseline (Week 0)				Post-intervention (Week 6)				Follow-up (Week 10)			Changes in scores TAU vs. COMB
													Week 0–6
		TAU (n = 22)	COMB (n = 23)	*d*		TAU (n = 22)	COMB (n = 21)	*d* (*p*)		TAU (n = 22)	COMB (n = 21)	*d* (*p*)	*d* (*p*)
AKQ-C													
	Mean (*SD*)	1.5 (1.6)	1.4 (1.3)	-0.04		1.7 (1.4)	5 (2.9)	1.47		0.9 (1.1)	5 (2.5)	2.14	1.51 (*p* < .0001)
AKQ-P													
	Mean (*SD*)	3.3 (1.8)	3.8 (1.7)	0.29		3.3 (1.5)	6.4 (3)	1.31		3.9 (2.1)	6 (2.3)	0.95	0.99
Vineland-II													
	Mean (*SD*)	61.4 (13.9)	63.5 (12.9)	0.16		61.6 (14.1)	70.3 (13.7)	0.63		62.6 (10.7)	70 (15)	0.56	0.6 (*p* = .0553)
SDQ													
	Mean (*SD*)	20.3 (5.5)	20 (4.7)	-0.05		21.1 (4.6)	17 (5.8)	-0.79		20 (4.5)	16.8 (5.7)	-0.61	-1.37 (*p* < .0001)
GHQ-12													
	Mean (*SD*)	17.1 (5)	16.4 (5.2)	-0.14		15.8 (5.8)	11.5 (5.2)	-0.78		16.6 (5.1)	12.7 (6.7)	-0.66	-0.61
DSRS-C													
	Mean (*SD*)	16.8 (8)	16.6 (7)	-0.03		14.9 (8.4)	14.3 (5)	-0.08		15.9 (8.7)	13.1 (5.5)	-0.39	-0.25 (*p* = .416)
BACE-3 C													
	Mean (*SD*)	25.7 (15)	24.1 (10.6)	-0.13		20.3 (13.4)	13.3 (8.7)	-0.62		18 (15)	12.7 (9.8)	-0.41	-0.46 (*p* = .1359)
BACE-3P													
	Mean (*SD*)	26.5 (10.7)	20.3 (11.8)	-0.55		26.2 (14.8)	20.1 (11.8)	-0.45		23.7 (12.6)	20 (9.5)	-0.33	-0.06
PREQ													
	Mean (*SD*)	79.9 (13.6)	83.4 (10.5)	0.29		80.3 (13.8)	87.9 (8.7)	0.65		80 (13)	87.3 (12.3)	0.58	0.52

AKQ-C, Autism Knowledge Quiz-Child; AKQ-P, Autism Knowledge Quiz-Parent; Vineland-II, Vineland Adaptive Behavior Scales Second Edition; SDQ, Strengths and Difficulties Questionnaire; GHQ-12, The 12-item General Health Questionnaire; DSRS-C, Depression Self-Rating Scale for Children; BACE-3 C, Barriers to Access to Care Evaluation scale, version 3 for children; BACE-3P, Barriers to Access to Care Evaluation scale, version 3 for parents; PREQ, Parenting Resilience Elements Questionnaire.

### Primary outcome

#### Adolescents’ self-awareness of their autistic traits

The primary outcome was self-awareness of autistic traits in autistic adolescents measured by the AKQ-C. The primary interest was to examine if there was a change in the AKQ-C score from pre- to post-intervention for the COMB group compared with the TAU group. After the intervention, comparing the two groups, the effect size of Cohen’s d, as 1.51 was more significant in the COMB group (Table 5).

Comparing an estimate of the effect size of η2 from the COMB group and TAU group using the analysis of covariance with Baseline AKQ-C scores and gender as covariates revealed that the change in the COMB group was larger than in the TAU group (p < .0001, η2 = 0.39) (Table S1). The estimate of the change from the baseline score was 0.2 in the TAU group (baseline: 1.5; week 6: 1.7) and 3.5 in the COMB group (baseline: 1.4; week 6: 5.0). These results indicated that the improvement in self-awareness of strengths and difficulties related to autistic traits was significantly greater in the COMB group than in the TAU group from pre- to post-intervention. Additionally, the values of Cohen’s d were 0.04 and 1.47 at each time point (pre- and post-intervention, respectively [Table 5]).

### Secondary outcomes

#### Awareness of children’s autistic traits among parents and guardians

The COMB group had a large effect size (d = 0.99), with scores increasing from 3.8 (SD = 1.7) at week 0 to 6.4 (SD = 3.0) at week 6. The COMB group also had a greater increase in AKQ-P scores than the TAU group.

#### Treatment stigma in autistic adolescents

We did not observe a change in treatment stigma in adolescents; the average score was 24.1 (SD = 10.6) at week 0 and 13.3 (SD = 8.7) at week 6. There were no differences in the degree of change in BACE-3 scores in the COMB group from week 0 to week 6 (d = -0.46, *p* = .1359).

#### Treatment stigma in parents/guardians

We did not observe a change in scores from 20.3 (SD = 11.8) at week 0 to 20.1 (SD = 11.8) at week 6.

#### General mental health, depression, and social adaptation in adolescents

There were differences between the COMB and TAU groups regarding the degree of change in general mental health; the average SDQ score in the COMB group reduced from 20 (SD = 4.7) at week 0 to 17 (SD = 5.8) at week 6. The COMB group showed a greater decrease in SDQ scores compared to the TAU group (*p* < .0001). However, there were no variations in the degree of change in social adaptive behavior, based on the Vineland-II, and in depression, based on the DSRS-C, from week 0 to week 6.

#### Mental health, adaptive skills, and attitudes toward children among parents/guardians

We did not observe a change in parents’ resilience; the average PREQ score was 83.4 (SD = 10.5) at week 0 and 80.3 (SD = 13.8) at week 6. The same was the case with the GHQ-12; we did not observe a change in average scores from 16.4 (5.2) at week 0 to 11.5 (5.2) at week 6 in the COMB group.

## Discussion

The objectives of this study were to evaluate the effectiveness of the ACAT intervention, developed to improve awareness of autistic traits among adolescents and parents/guardians. This would reduce treatment stigma and improve adolescents’ coping skills and social adaptation. We hypothesized that: (1) adolescents and parents/guardians in the COMB group would show improved awareness of autistic traits compared to those in the TAU group; (2) adolescents and parents/guardians in the COMB group would show reduced treatment stigma compared to those in the TAU group; (3) adolescents and parents/guardians in the COMB group would show decreased depression and psychological distress levels, and increased social adaptation, compared to those in the TAU group. As expected, the ACAT program increased awareness of autistic traits both among adolescents and their parents/guardians. In addition, it reduced treatment stigma and negative emotional and behavioral attributes in autistic adolescents. However, there were no effects on adolescents’ adaptive behavior and depression, as well as on treatment stigma, mental health, adaptive skills, and attitudes toward their children among parents/guardians.

The ACAT was designed to improve the metacognition of cognitive and behavioral patterns for autistic adolescents by adding the “awareness of autistic traits” to CBT. With this approach, the metacognition of cognitive and behavioral patterns, which is often attenuated in autistic individuals and is difficult to conceptualize in the CBT model, can be targeted.

### Improved awareness regarding autistic traits in adolescents

The improvement in adolescents’ self-awareness regarding their autistic traits and understanding of their children’s autistic traits among parents/guardians was significantly greater in those who participated in the ACAT program than in those who received TAU. This suggests that the ACAT was effective in helping autistic adolescents and their parents/guardians become aware of the former’s autistic traits, particularly their strengths. CBT involves cognitive and behavioral procedures to understand one’s response patterns through “metacognition,” which is the process of monitoring one’s responses in problem-solving and transforming those response patterns [33]. In the ACAT program, for children and adolescents, an awareness of the difficulties and strengths associated with their autistic traits is imperative for better awareness of their response patterns.

### Reduced treatment stigma in adolescents

Treatment stigma was reduced in adolescents in both the COMB and TAU groups. Autism diagnosis may be useful if those who receive it have adequate knowledge and self-understanding of autistic traits, which could improve access to services, help formulation of autistic identity, and guide future planning. However, stigmatization and lower self-esteem [6] may inhibit help-seeking or treatment-seeking behaviors. Additionally, owing to stigma, children and adolescents with autism may feel ashamed and believe that they are bothering others, making them less likely to request reasonable accommodations [34]. In this case, social maladjustment is likely to occur. A study revealed that adolescents whose parents voluntarily disclosed their autism diagnosis and were able to discuss autism were more willing to explain themselves and their autism than those who did not. Furthermore, they were believed to have a more neutral self-identity, rather than identifying as “disabled people,” as suggested by the medical model [11, 35]. These findings also supported our working hypothesis that the ACAT contributed to reducing stigma by increasing recipients’ knowledge and awareness of autistic traits.

### Improvements in general mental health among autistic adolescents

Based on the SDQ scores, in the COMB group, there were improvements in adolescents’ general mental health, but this was not the case with the TAU group. Furthermore, based on the Vineland-II, the social adjustment did not significantly improve in autistic adolescents, but the effect size was moderate (d = 0.60). These findings suggest that the ACAT program was effective for the general mental health of autistic adolescents but not for their social adjustment. Contrastingly, the change in the score of depressive symptoms was not significantly different between the COMB and TAU groups, and the effect sizes were low (d = 0.03, d = 0.08). The average DSRS-C score in the COMB and TAU groups was 16.8 and 16.6, respectively, below the cut-off of 19.3 for current depression [36]. The prevalence rates of depression in autistic children are inconsistent in published studies, ranging from 6 to 26% [37, 38]. This intervention did not target autistic adolescents with comorbid depression, which may explain the lack of change in depression indicators before and after the intervention.

Employing the therapeutic process of CBT, cognitive and behavioral strategies can be created through increased awareness of problems (metacognition and cognition) [33]. As we used CBT in the ACAT program, a more functional coping strategy for autism-related maladjustment can be developed through increased awareness of the characteristics of autistic traits. We expect that the mental health and social adjustment of autistic individuals will improve by applying a more functional coping strategy in their daily lives. However, it may take longer to improve social adjustment, and a one-month follow-up might not be enough to observe the improvement. Nevertheless, our results confirmed that the ACAT program effectively reduced negative behavioral attributes and improved autism awareness in autistic adolescents, suggesting that the CBT component in the ACAT program could be effective in a series of treatment processes.

### Improved awareness of autistic traits in parents/guardians

The increase in awareness of their children’s autistic traits among parents/guardians (AKQ-P) between week 0 and week 6 was significantly higher in the COMB group than in the TAU group. This indicates that the ACAT program can help parents/guardians understand and recognize their children’s autistic traits. However, in the COMB group, parents/guardians’ mental health, stigma-related barriers to treatment-seeking behaviors, and parenting resilience did not significantly improve. Parents of autistic children tend to experience more stress and are more susceptible to negative outcomes than parents of children with other disabilities [39]. Although the ACAT program helped parents/guardians understand their children’s autistic traits, it had not, throughout the study, decreased treatment stigma, improved mental health, or improved parenting resilience among parents/guardians. Factors other than awareness of their children’s autistic traits might be relevant, and we may need to include additional support strategies or booster sessions for parents/guardians in the ACAT program. For autistic adolescents, the ACAT program improved self-understanding and negative behavioral attributes and reduced barriers to receiving support; however, it did not reduce depression or improve social adjustment. Meanwhile, for parents/guardians, the ACAT program improved their understanding of their children’s autistic characteristics but did not reduce barriers to obtaining support for their autistic children; furthermore, it did not improve their mental health or parenting resilience. Based on these results, we infer that the ACAT program may positively affect self-understanding and reduce treatment stigma and stabilize behavioral issues for autistic adolescents, but it may not effectively reduce treatment barriers and improve mental health for parents/guardians. How the combined program aspect of ACAT (i.e., adolescents and parents/guardians attend the session together) enhances its effect on reducing treatment stigma and psychological burdens should be further explored. Further research should consider whether a combined program is the best for reducing treatment stigma and improving the mental health of both autistic adolescents and their parents/guardians, or whether additional individual sessions for parents/guardians could be beneficial. Therefore, we suggest using ACAT as an effective support program for autistic adolescents to reduce their barriers to treatment-seeking behaviors, by increasing autism awareness both in adolescents and their parents/guardians.

### Limitations

This study has several limitations. First, as the sample size was small and diagnostic timing was not considered, we did not control for the presence of other mental disorders. Second, when comparing the ACAT program and TAU, it was unclear which aspects of the former contributed to changes in self-awareness of autistic traits (both in adolescents and parents/guardians) and treatment stigma (only in adolescents). As a next step, we need to conduct a randomized controlled trial of the ACAT program vs. PEGASUS. Third, the study was designed for autistic adolescents with an IQ of ≥ 90 on the VCI, which limits its general versatility. Fourth, we excluded the parents/guardians of autistic adolescents who met the diagnostic criteria for a psychiatric disorder. Parents of autistic youth are more likely to have a psychiatric diagnosis than parents of non-autistic youth and thus, this decision limits the generalizability of the findings to parents/guardians of autistic adolescents. Fifth, the fidelity of the intervention was only measured for about 4% of the total sessions (i.e., 12 of 270 sessions), lower than the standard 10–20% range for intervention studies and thus constitutes a limitation. Sixth, only parents/guardians with no psychiatric disorders were included in the study, and therefore, there is little to no understanding of the intervention’s use in real clinical situations where both children and parents/guardians have autistic traits. Finally, we aimed to clarify the effects of autism awareness and coping skills on stigma and mental health. However, as coping skills were not directly measured, future studies should aim to do this.

## Conclusions

The ACAT program is effective in improving autistic adolescents’ self-awareness of autistic traits, and it can help parents/guardians understand their adolescents’ autistic traits. It also helps improve general mental health and reduce treatment stigma in autistic adolescents. Although we did not observe significant effects on depression and social adaptation for autistic adolescents and treatment stigma, mental health, and parenting resilience for parents/guardians, the ACAT program was useful for improving parent-child communication and an understanding of children’s autistic traits. The effect on parents/guardians was less than that on adolescents. Therefore, improving the intervention for parents/guardians should be considered in the future. Further longitudinal longer-term trials are needed to determine whether the ACAT program can enhance adaptive social behaviors and prevent subsequent mental health issues, such as depression, anxiety, and low self-esteem, for both autistic children and their parents/guardians.

### Electronic supplementary material

Below is the link to the electronic supplementary material.

Supplementary Material 1: Supplementary materials for Awareness and Care for my Autistic Traits (ACAT) program for autistic adolescents: a multicenter randomized controlled trial

## Data Availability

The datasets used and/or analyzed during the current study are available from the corresponding author upon reasonable request.

## List of abbreviations

ADI-R: Autism Diagnostic Interview-Revised
ADOS-2: Autism Diagnostic Observation Schedule
AKQ-C: Autism Knowledge Quiz-Child
AKQ-P: Autism Knowledge Quiz-Parents
ACAT: Awareness and Care for my Autistic Traits
BACE-3: Barriers to Access to Care Evaluation scale version 3
CBT: Cognitive-behavioral therapy
COMB group: Combination of awareness and care for my Autistic Traits and treatment-as-usual
DSRS-C: Depression Self-Rating Scale for Children
GHQ-12: General Health Questionnaire
IQ: Intelligence quotient
PREQ: Parenting Resilience Elements Questionnaire
CTS-R: Revised Cognitive Therapy Scale
SDQ: Strengths and Difficulties Questionnaire
TAU: Treatment-as-usual
VCI: Verbal Comprehension Index
WISC-IV: Wechsler Intelligence Scale for Children, Fourth Edition
