# Supplementary material for: Cognitive behavior therapy for autistic adolescents, awareness and care for my autistic traits program: a multicenter randomized controlled trial
Source: BMC Psychiatry. 2023 Sep 7;23:661. doi: 10.1186/s12888-023-05075-2 (PMC10485995; doi:10.1186/s12888-023-05075-2)
Supplement: Supplementary file 1 — Supplementary Material 1: Supplementary materials for Awareness and Care for my Autistic Traits (ACAT) program for autistic adolescents: a multicenter randomized controlled trial [file 12888_2023_5075_MOESM1_ESM.docx]

Supplementary materials for

**Awareness and Care for my Autistic Traits (ACAT) program for autistic adolescents: a multicenter randomized controlled trial**

Fumiyo Oshima^1,2,*^, William Mandy^3^, Mikuko Seto^1^, Minako Hongo^1,2^, Aki Tsuchiyagaito^4^, Yoshiyuki Hirano^1,2^, Chihiro Sutoh^6^, Masaru Kuno^1,2^, Siqing Guan^1^, Yusuke Nitta^1^, Yoshihito Ozawa^6^, Yohei Kawasaki^6^, Toshiyuki Ohtani^2,9^，Daisuke Matsuzawa^1^, Jiro Masuya^7^, Noriko Takahashi^8^, Noriyuki Sato^8^, Shizuka Nakamura^8^, Akiko Nakagawa^1,2^ and Eiji Shimizu^1,2^.

^1^ Research Center for Child Mental Development, Chiba University, 1-8-1 Inohana, Chuouku, Chiba 260-8670, Japan

^2^ Division of Cognitive Behavioral Science (Chiba University), United Graduate School of Child Development, Osaka University, Kanazawa University, Hamamatsu University School of Medicine, Chiba University and University of Fukui, Japan

^3^ Research Department of Clinical, Educational & Health Psychology, University College London, Gower Street, London, WC1E 6BT, UK

^4^ Laureate Instituto for Brain Research, 6655 S Yale Ave, Tulsa, OK, 74136, USA

^5^ Department of Cognitive Behavioral Physiology, Chiba University, 1-8-1 Inohana, Chuouku, Chiba 260-8670, Japan

^6^ Biostatistics Section, Clinical Research Center, Chiba University Hospital, Chiba University, 1-8-1 Inohana, Chuouku, Chiba 260-8670, Japan

^7^ Department of Psychiatry, Tokyo Medical University Ibaraki, Medical Center, 3-20-1 3-20-1 Chuo, Ami-machi, Inashiki-gun, Ibaraki 300-0395, Japan

^8^ Fukushima University Child Mental Health-Care Center, 1 Kanayagawa, Fukushima 960-1296, Japan

^9^ Safety and Health Organization, Chiba University, Chiba, Japan.

***Corresponding author**: Fumiyo Oshima. Tel: (+81)-43-226-2027; Fax: (+81)-43-226-2028; Email: f_oshima@chiba-u.jp.

**Content:**

**Supplementary Method**

Scoring of Autism Knowledge Quiz-Child (AKQ-C) and Autism Knowledge Quiz-Parent (AKQ-P) ------ Page 3

**Supplementary Result**

**Table of F value of ANCOVA** ------ Page 4

**Supplementary Figure**

Figure S1 ------ Page 5

Figure S2 ------ Page 6

**Supplementary Method**

**Scoring of Autism Knowledge Quiz-Child (AKQ-C) and Autism Knowledge Quiz-Parent (AKQ-P)**

To quantitatively measure an individual's autistic awareness, the interviewer, an independent evaluator with no visible grouping, asked two standard questions using prompts (e.g., "Strengths are what a person is good at. What are your strengths?") In response, respondents provided verbal explanations. The first question did not mention the word "autistic traits" in the question text, and the subsequent question asked, "What are your strengths, as they relate to your autistic traits? " The second question used the term "autistic traits" for the prompt. Likewise, it asked, "What are your weaknesses?" and the subsequent question asked, "What are your weaknesses as they relate to your autistic traits?"

The authors who developed the Psychoeducation Group for Autism Spectrum Understanding and Support (PEGASUS) (Gordon et al., 2015) developed the AKQ-C and AKQ-P. The AKQ-C is scored as 1 if the verbal expressions are associated with an autistic trait, such as "I am never bored" or "I can focus on what I like," and 0 if they are not associated with an autistic trait, such as, "I am good at playing soccer."

The first and second questions are scored similarly, indicating that the questions are looking for autistic-related weaknesses/strengths whether respondents are directly asked about it. In addition, Gordon et al. (2015) list which free-text statements can be counted as autistic traits. We have used the notations in the list (e.g., positive effects of concrete thinking/thinking in a rule-based/logical way, sticking to the rules/doing the right thing/moralistic/ keeps promises/reliable/sense of fairness, etc.) and scored them. The co-author W.M. guided our scoring.

**Reference:** Gordon, K., Murin, M., Baykaner, O., Roughan, L., Livermore-Hardy, V., Skuse, D., & Mandy, W. (2015). A randomized controlled trial of PEGASUS, a psychoeducational program for young people with high-functioning autism spectrum disorder. *Journal of Child Psychology and Psychiatry, and Allied Disciplines*, *56*(4), 468–476. https://doi.org/10.1111/jcpp.12304

**Supplementary Table**

The supplementary table shows the ANCOVA results for primary analysis (F statistics) – for main and follow-up analyses (Table S1).

Table S1. The primary analysis of AKQ-C by covariates

| (A) |  |  |  |  |
| --- | --- | --- | --- | --- |
| Source | df | F value | Pr>F |  |
| Model ^*)^ | 3 | 9.27 | <.0001 |  |
| Error | 39 |  |  |  |
| Total | 42 |  |  |  |
| (B) |  |  |  |  |
| Factor | df | F value | Pr > F | η^2^ |
| Group ( TAU vs COMB) | 1 | 25.36 | <.0001 | 0.39 |
| Gender | 1 | 0.47 | 0.4991 | 0.01 |
| Baseline | 1 | 2.35 | 0.1337 | 0.05 |

(A) ANCOVA table

(B) Details for models ^*)^ listed in (A) are shown in the table.

Group as Primary comparison

Gender and Baseline as Covariance

**Supplementary Figures**


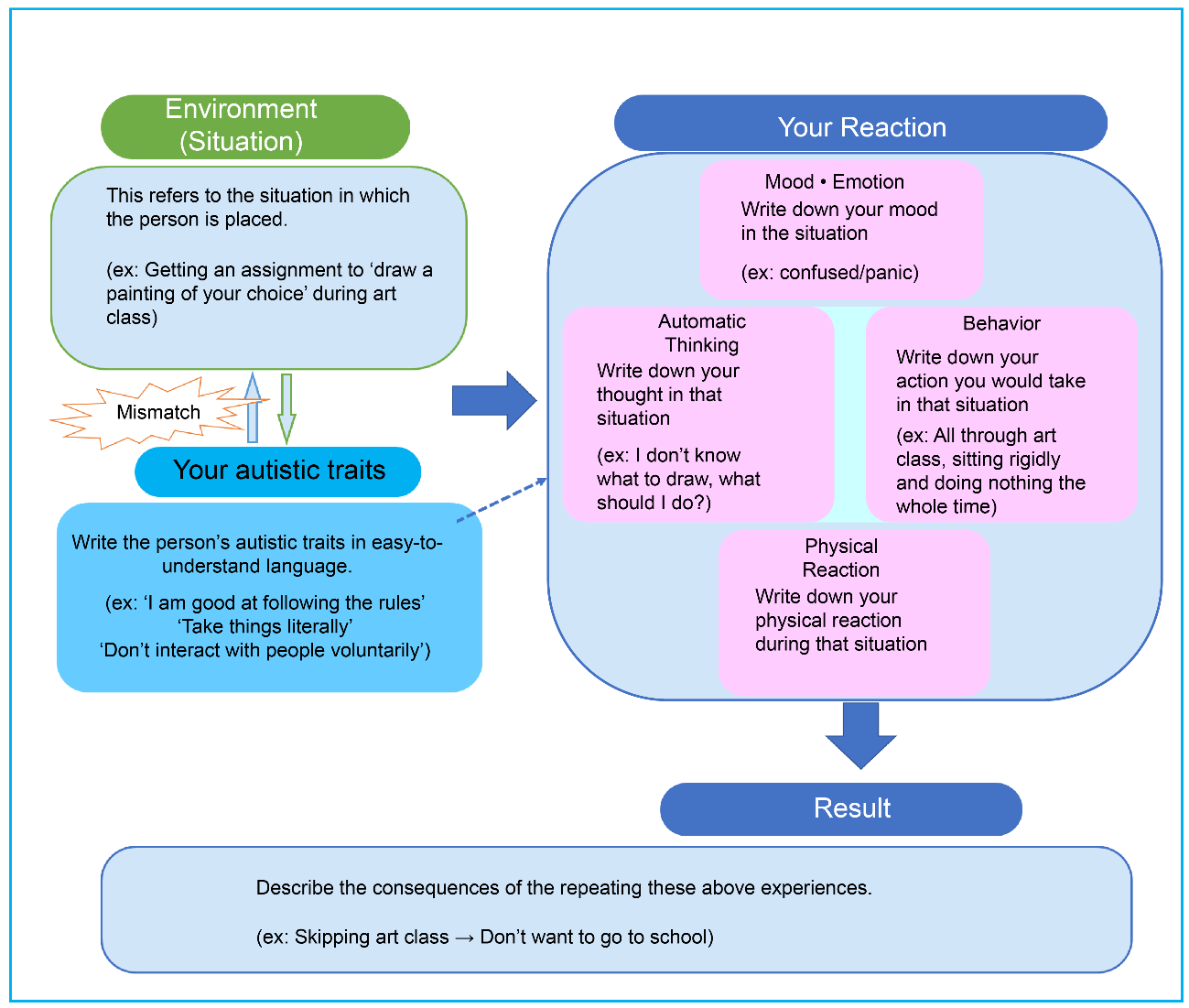


**Figure S1.** An example of the CBT model used in the ACAT to understand an adolescent’s autistic traits


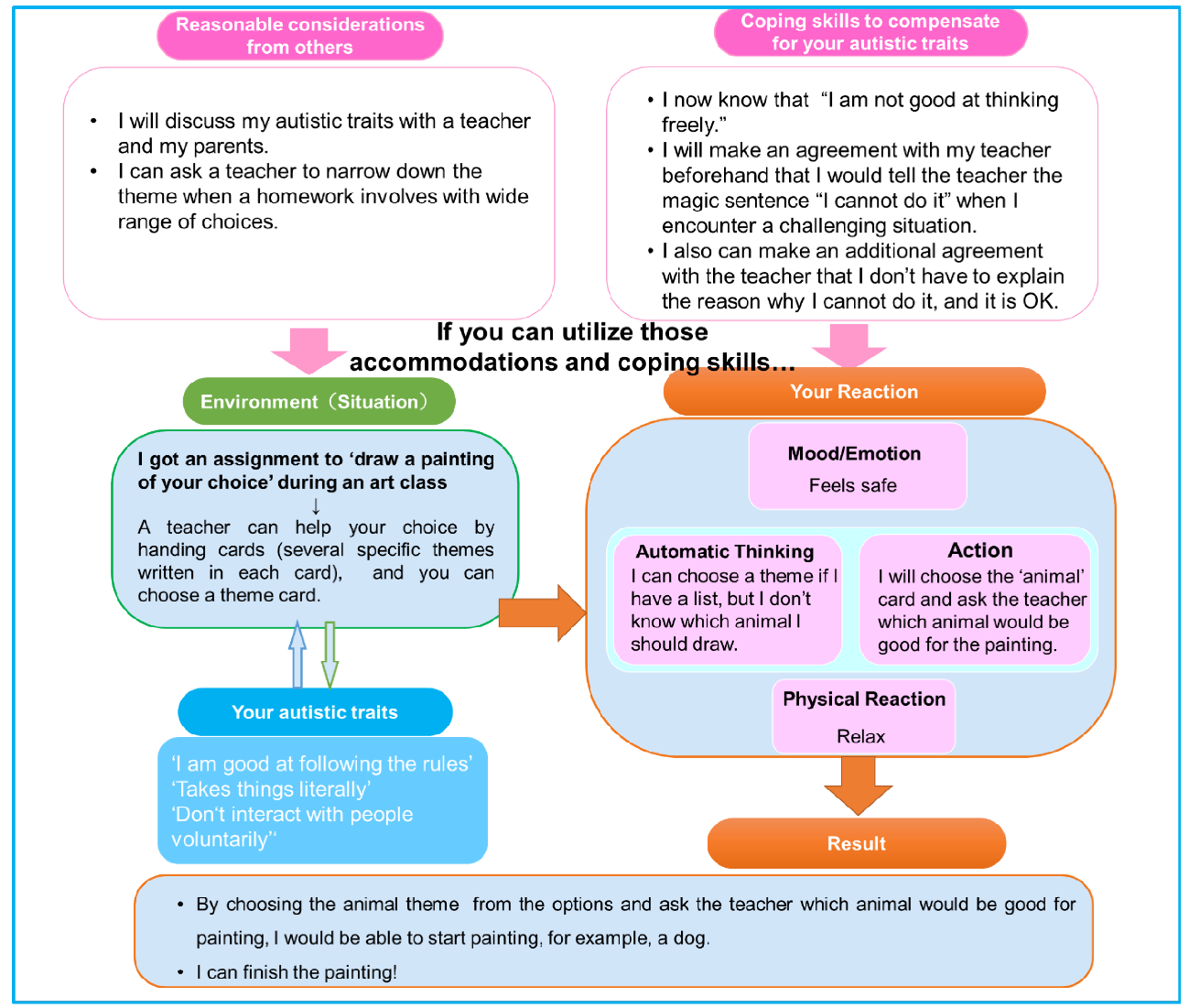


**Figure S2.** An example of brainstorming work used in the ACAT to come up with reasonable accommodations and coping skills
